# Study of the Molecule Adsorption Process during the Molecular Doping

**DOI:** 10.3390/nano11081899

**Published:** 2021-07-24

**Authors:** Mattia Pizzone, Maria Grazia Grimaldi, Antonino La Magna, Neda Rahmani, Silvia Scalese, Jost Adam, Rosaria A. Puglisi

**Affiliations:** 1 Istituto per la Microelettronica e Microsistemi (IMM), Consiglio Nazionale delle Ricerche (CNR), Strada Ottava 5, Zona Industriale, 95121 Catania, Italy; mattia.pizzone@phd.unict.it (M.P.); antonino.lamagna@imm.cnr.it (A.L.M.); silvia.scalese@imm.cnr.it (S.S.); 2 Dipartimento di Fisica e Astronomia “Ettore Majorana”, Università degli Studi di Catania, Via S. Sofia, 64, 95123 Catania, Italy; mariagrazia.grimaldi@ct.infn.it; 3 Computational Materials Group, Department of Mechanical and Electrical Engineering (DME), University of Southern Denmark (SDU), DK-6400 Sønderborg, Denmark; neda@sdu.dk (N.R.); jostadam@sdu.dk (J.A.)

**Keywords:** self-assembled monolayer, monolayer doping, molecular doping, liquid phase deposition, surface treatments, density functional theory, doping profile

## Abstract

Molecular Doping (MD) involves the deposition of molecules, containing the dopant atoms and dissolved in liquid solutions, over the surface of a semiconductor before the drive-in step. The control on the characteristics of the final doped samples resides on the in-depth study of the molecule behaviour once deposited. It is already known that the molecules form a self-assembled monolayer over the surface of the sample, but little is known about the role and behaviour of possible multiple layers that could be deposited on it after extended deposition times. In this work, we investigate the molecular surface coverage over time of diethyl-propyl phosphonate on silicon, by employing high-resolution morphological and electrical characterization, and examine the effects of the post-deposition surface treatments on it. We present these data together with density functional theory simulations of the molecules–substrate system and electrical measurements of the doped samples. The results allow us to recognise a difference in the bonding types involved in the formation of the molecular layers and how these influence the final doping profile of the samples. This will improve the control on the electrical properties of MD-based devices, allowing for a finer tuning of their performance.

## 1. Introduction

Doping of semiconductors, i.e., the introduction of dopant impurities into the intrinsic material to modulate its electrical properties, is one of the main steps in device fabrication. Conventional doping methods (ion implantation and diffusion based methods) present issues related to cost, safety, crystal lattice damage, and difficulties in obtaining conformal doping profiles, essential in nano-device fabrication, especially when non planar architectures are used. Monolayer Doping, also known in the literature as Molecular Doping (MD), has been demonstrated as a low-cost alternative to conventional doping techniques [1,2]. Differently from another literature process, where guest molecules—working as charge transfer layer—are put in contact with the host material [3,4,5,6,7], the method involves the deposition of dopant-containing molecules from the liquid phase, called precursors, and the subsequent drive-in of the dopant atoms by thermal annealing. MD can provide n- and p-type doping and is capable of a good range of carrier doses and diffusion depths, obtained by controlling the precursor chemical characteristics, the deposition conditions, and the thermal budget [1,2,8,9,10,11,12,13]. In the work of Javey and their group [1], during the MD process, the Si substrate is immersed in a solution containing the molecular precursor, kept at its boiling point, for 150 min, during which a self-assembled monolayer of molecules is formed on the sample surface. The sample is then subjected to an annealing process. In this step, the source molecules decompose, and the dopant diffuses into the substrate lattice, with kinetics typical of the diffusion phenomenon. It is suggested that the molecule, during the deposition, forms a covalent bond with the Si surface, involving its C=C site [1]. Since then, MD has been successfully used for the deposition of molecular precursors and diffusion in both planar and nano-structured surfaces with no crystal lattice damage [13,14,15,16,17]. Many of the results present in the literature refer to a 20% dilution condition. The control of the dopant dose has also been studied by an approach where the precursor has been diluted with a neutral molecule not participating in the doping process [18]. In the literature it has been proposed to cover the source molecules with a silicon oxide cap layer to prevent evaporation during the thermal annealing, although it has been shown in a successive work that the process is effective also without the cap [19]. Preliminary investigations on the process efficiency have been performed. Metallurgical junctions with depths as small as 5 nm and 10 nm, respectively, for n+ and p+ doping, with doping concentrations of the order of 10${}^{20}$ cm${}^{-3}$ have been demonstrated [20,21,22,23]. MD has also been used for the realization of Si nanowire-based solar cells [2] and as a valid doping technique for many geometries and other applications [9,10,17,24]. Furthermore, it has been used as a scalable technique for industrial applications on a 4 inches Si wafer [20]. Although the most used substrate to be doped by MD is Si [14,25,26], other substrates have been used, such as InAs, InGaAs, oxidized silicon, alumina, and mica [27,28,29,30]. Other precursors, such as OPA molecules, have also been explored on different substrates [31,32,33,34]. These results show the potential to control the MD processing. Although the investigation of molecular multi-layer formation during the deposition has been performed in the past [35,36], a systematic study on their formation has not been presented so far. Direct observation at high-resolution of the deposited molecular layers evolution has neither been done. This work shows the direct observation of the molecules morphology over time, the multi-layer formation, and the effects of post-deposition treatments on them. We study the surface coverage of Diethyl-Propyl Phosphonate (DPP) at 10% dilution and the morphology of the aggregates over the Si substrate by Scanning Electron Microscopy (SEM). We corroborate these results with Density Functional Theory (DFT) simulations of the grafted molecule over Si, which has been observed in a previous work [37]. We present these data together with electrical measurements of carrier concentration depth profiles produced with the MD process at different synthesis conditions by Spreading Resistance Profiling (SRP).

## 2. Materials and Methods

### 2.1. Experimental Methods

Si samples, 1×1 cm${}^{2}$ 1–10 ohm/sq <111> p-type, have been employed for this study. Prior to the molecule deposition, the samples have been cleaned in HF. The molecule deposition step has been carried out in a 10% concentrated (*v*/*v*) DPP (Abcr, 95%) and Mesitylene (Abcr, 98+%), kept at the solution boiling temperature for the desired time, ranging from 7 to 150 min. The samples have been divided into two groups, one to be analyzed right after the deposition (“as-deposited” samples), the other to be cleaned with a surface treatment consisting in rinsing in acetone, ethanol, water and stirring in acetone with a magnetic stirrer for 15 min (“cleaned” samples).

Scanning Electron Microscope (SEM) analysis was performed by a Zeiss Supra 35 FE-SEM (Oberkochen, Germany), with a primary energy beam of 3keV. From the SEM images we extracted the surface coverage data by calculating fast fourier transforms (FFT) of the images, applying a proper bandpass filter mask on it, and calculating the inverse FFT of the filtered transforms obtained [38]. This method ensures a noise reduction of the images and allows for the recognition of the areas to be measured by determining a threshold in the electron intensity spectrum distribution over which the areas are recognized and measured. All measurements and calculations have been done with Digital Micrograph 3. The image contrast has been optimized to increase the signal-to-noise ratio. The images resolution allowed for the recognition of clusters down to 32 nm${}^{2}$ (6.4 nm diameter, assuming clusters as circular).

The annealing process was performed at 1050 °C for 500 s in N_2_ environment, with a 45 min ramp of 10 °C per minute, starting from 600 °C. No oxide layer has been deposited on the samples prior to the annealing step.

Spreading Resistance Profile (SRP) data were collected to calculate the carrier dose of a few samples. The technique consists of using two metallic probes to measure a resistance profile of a beveled sample with an electric potential of 5 mV. Resistance is converted into resistivity and, with the use of calibration curves, carrier concentration and carrier dose can be calculated. SRP measurements have been carried out by a SSM150 tool.

### 2.2. Computational Methods

The simulations have been performed using density functional theory (DFT) implemented in the SIESTA code [39]. We employed the generalized gradient approximation (GGA) as formulated by Perdew–Burke–Ernzerhof (PBE) to approximate the exchange-correlation functional [40]. We applied the Monkhorst–Pack scheme [41] with 7×7×1 k-points for sampling the Brillouin zone and a mech cut-off 300 Ry for the calculations. The atomic positions were optimized until the Hellmann–Feynman forces on the atoms were less than 0.05 eV/Å and the energy convergence threshold was 1e−4 eV. We have modeled a silicon (111) surface with a 2×1 reconstruction using a slab of 24 atoms with 6.60 Å and 7.70 Å lattice parameters and 18.06 Å of the vacuum region to avoid interlayer interaction. The dangling bonds on the bottom surface have been passivated with hydrogen to prevent charge migration.

## 3. Results and Discussion

**Figure 1:**
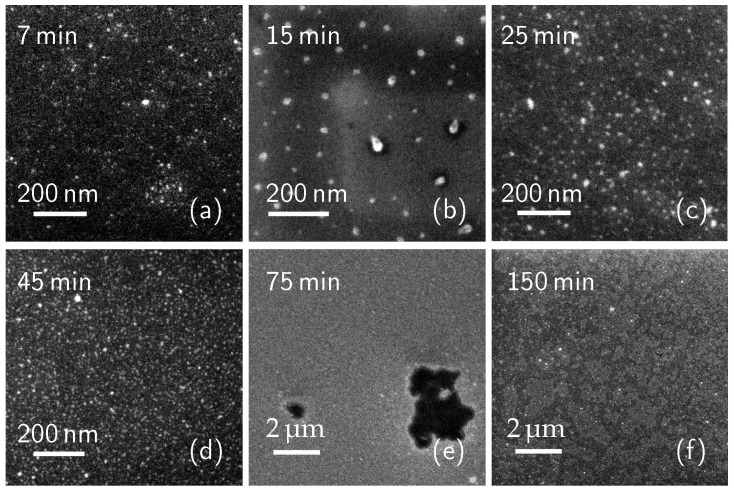
SEM plan images of the as-deposited samples with a deposition time of 7 (**a**), 15 (**b**), 25 (**c**), 45 (**d**), 75 (**e**), 150 (**f**) minutes. Image (**e**) shows the molecular layer next to completion, while image (**f**) exhibits the presence of molecular terraces.

The investigation of the deposition step has been conducted in a 10% concentrated (*v*/*v*) solution of DPP and Mesitylene, kept at its boiling point. The samples were immersed inside the solution and taken out after 7, 15, 25, 45, 60, 75, 120 and 150 min. After the deposition step, some of the samples did not undergo any other treatment (“as-deposited” samples), while the others went through a surface cleaning process (“cleaned” samples) consisting in rinsing with acetone, alcohol and water, and 15 min magnetic stirring in acetone. We analyzed both sample groups by SEM. Figure 1 shows the SEM plan images of the as-deposited samples with a deposition time of 7 (**a**), 15 (**b**), 25 (**c**), 45 (**d**), 75 (**e**), 150 (**f**) minutes. The surface coverage of each of these samples was measured over several images and an average value was calculated.

**Figure 2:**
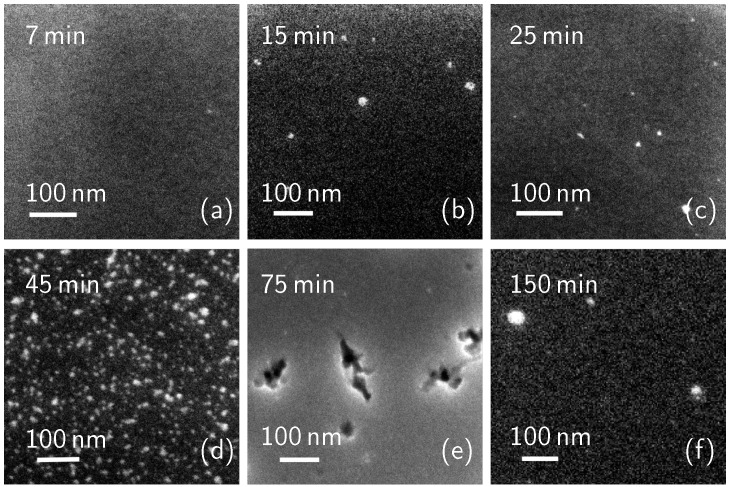
SEM plan images of the cleaned samples at (**a**), 15 (**b**), 25 (**c**), 45 (**d**), 75 (**e**), 150 (**f**) minutes.

As it can be seen in Figure 1a–d, the molecule cluster surface density grows as deposition time increases. The calculated surface coverage is 2.2% (7 min sample), 5% (15 min), 4.9% (25 min), and 15.4% (45 min), showing a significant increase between 25 and 45 min. At 75 min (Figure 1e), only a few dark areas can be seen. The measured surface coverage is 88.3%. From this data, we can estimate a 100% coverage value at about 100–120 min. The surface of the sample dip-coated for 150 min can be seen in Figure 1f. The image presents bright regions corresponding to terraces, which could be seen as the first layer of DPP molecules, and the second layer of molecule terraces on top of the first. The surface coverage of this sample is then 149%, indicating that there is a full, 100% coverage first layer of molecules directly over the Si surface and the second layer of molecules covering 49% of the first layer surface area. These data will be discussed later, together with the carrier concentration profile measurements. While the presence of multi-layers and their removal from the top of the sample has been mentioned in many previous works [1,42,43], a deep investigation of the cleaning process effects on the molecule layers has not been done yet. In this work, the effects of surface cleaning on the molecule coverage have been investigated by cleaning a group of the produced samples right after the deposition step. Figure 2 shows the SEM plan images of the cleaned samples with a deposition time of 7 (**a**), 15 (**b**), 25 (**c**), 45 (**d**), 75 (**e**), 150 (**f**) minutes. Again, the molecule surface coverage has been evaluated.

**Figure 3:**
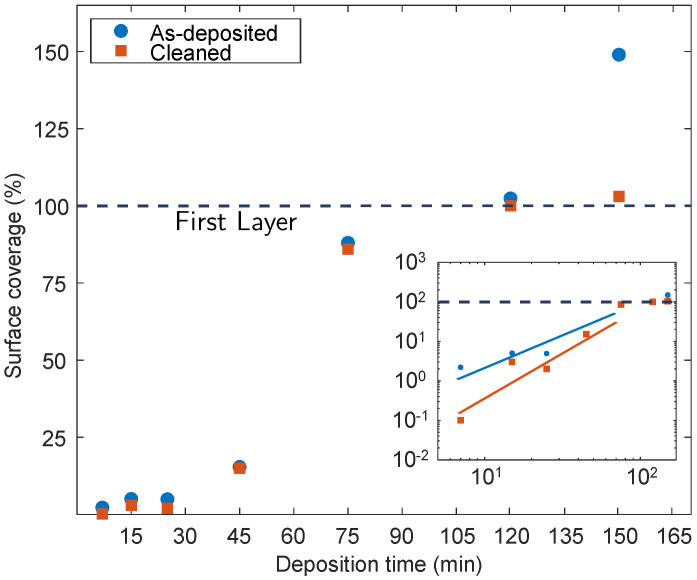
Molecule surface coverage vs. deposition time. Coverage values over 100% indicate the completion of the first molecular layer and the growth of a second layer on top of the first one. Inset: log-log chart of the same data. Blue and orange lines are a guide to the eye.

Despite the cleaning process, from the analysis, we observe that all the samples present the molecules on their surface, even though the surface density is lower with respect to the as-deposited samples, especially for the low deposition times. Evidently, these molecules have not been removed by the surface cleaning process, which can probably be attributed to the covalent nature of their bonds with the Si surface. The sample dip-coated for 7 min (Figure 2a) presents a surface coverage of 0.3%. Figure 2b (15 min) and Figure 2c (25 min) have a very similar morphology: both of these samples present sparse molecule nucleation spots, covering 3% and 2%, respectively. Figure 2d,e show a SEM plan image of a dip-coated sample for 45 and 75 min, respectively. The calculated surface coverage is 15% (45 min) and 86.2% (75 min), values in accordance with the as-deposited samples (Figure 1d,e) within experimental errors. Figure 2f shows the surface morphology of a 150 min dip-coated and cleaned sample. In this image, we can see that the surface appears mostly flat, with some molecule clusters with a bright appearance. The surface coverage of this sample is 103%, accounting for a 100% coverage molecule layer, plus a 3% coverage of the molecule clusters over the first layer. Also these data will be discussed below. Surface coverage data from both sets of samples are represented in the graph of Figure 3. In this image, blue dots represent data from the as-deposited samples, while orange squares represent coverage of the cleaned samples. Coverage over 100% indicates the completion of the first layer and the deposition of a second layer on top of the first.

From the data in Figure 3, it can be deduced that at the beginning of the deposition process, in particular up to the 25th minute, the deposition develops slowly. During these early stages, the surface coverage barely changes and, within experimental errors, tends to slightly increase, between 7 and 25 min. At 45 min, we can see that the curve starts to rise. The growth rate escalates between 45 and 75 min. From the comparison between the as-deposited and the cleaned samples, blue and orange data, we can deduce information on the bond type between the aggregates and the substrate. As it is evident, after the surface cleaning, the coverage drops, which could be representative of these bond types: covalent strong bonds and Van der Waals weak forces. Aggregates can be observed in both as-deposited and cleaned samples, even for low deposition times. Since rinsing processes hardly remove chemically bonded molecules, we can take these surface coverage measurements as an indirect quantitative evaluation of existing covalent bonds at the investigated deposition times. The data at 45 min show that both the as-deposited and the cleaned samples present similar surface coverage within experimental errors. This could be an indicator of the fact that most of the present molecules in the as-deposited sample are chemically bonded. This explains the early stage of the deposition, in both cleaned and as-deposited conditions. The rapid increase in coverage, from the 45th minute on, is probably due to a switch in deposition regime: in the first phases of the deposition, the creation of new molecular clusters occurs (nucleation phase), and the coverage increases slowly, while in the second part the created clusters tend to grow (growth phase) as described by the Avrami model [44]. When the second growth regime starts, molecules will also contribute to the growth of the pre-existing clusters. For the time interval 45–120 min the ratio between covalently bonded and physically bonded molecules becomes larger, hence we observe the shrinkage of the difference in surface coverage between as-deposited and cleaned samples. After 120 min, as the first layer becomes complete, a second molecule layer starts to form on top of it. These newly deposited molecules will not be able to bond with the Si surface and will be physisorbed instead. The subsequent surface treatment removes the new layers: this explains the difference between cleaned and as-deposited samples in the latter parts of the investigated growth time. Nevertheless, further data and investigation are needed to understand more about the second layer formation. The inset in Figure 3 shows the log-log chart of surface coverage data, with the lines drawn as a guide to the eye.

**Figure 4:**
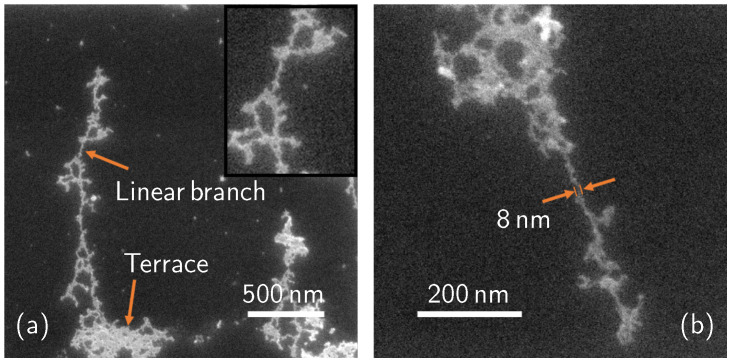
SEM plan images of the samples dip-coated in a 10% concentrated DPP solution for 15 min and cleaned after the deposition step. (**a**) Molecular branch and terrace, the inset shows a detail of the linear branch. (**b**) A thin, developed branch.

Figure 4 reports an example of the molecular complexes formed on the samples obtained after 15 min deposition and cleaning treatment. These structures are observable as well in the 45 min treated samples. The surface cleaning has probably uncovered the shape of these structures since physisorbed material could have deposited on top of them in an unshapely fashion, making it very difficult for the instruments to catch the detailed morphology underneath. We then speculate that, as described before, these structures are covalently bonded to the substrate. In Figure 4a, two types of structure can be distinguished: terraces and linear branches. The minimum measured thickness of the linear branches is 8 nm, as indicated in Figure 4b.

To better understand these features and how the molecules are arranged inside them, we performed DFT calculations (see the Methods section for technical details), providing information on the possible bond configurations. We covered the Si (111) 2×1 reconstructed surface by four broken DPP molecules in a way that three oxygens of the molecules initially bond with silicon atoms. The DPP molecule has been modeled without the two lateral aliphatic chains (-CH${}_{2}$-CH${}_{3}$) because we expect such a decomposition from previous Raman measurements performed on the samples after the molecule deposition [10]. It is not clear when the molecule loses these chains: it could happen either inside the solution or due to the interaction between the molecule and the substrate.

**Figure 5:**
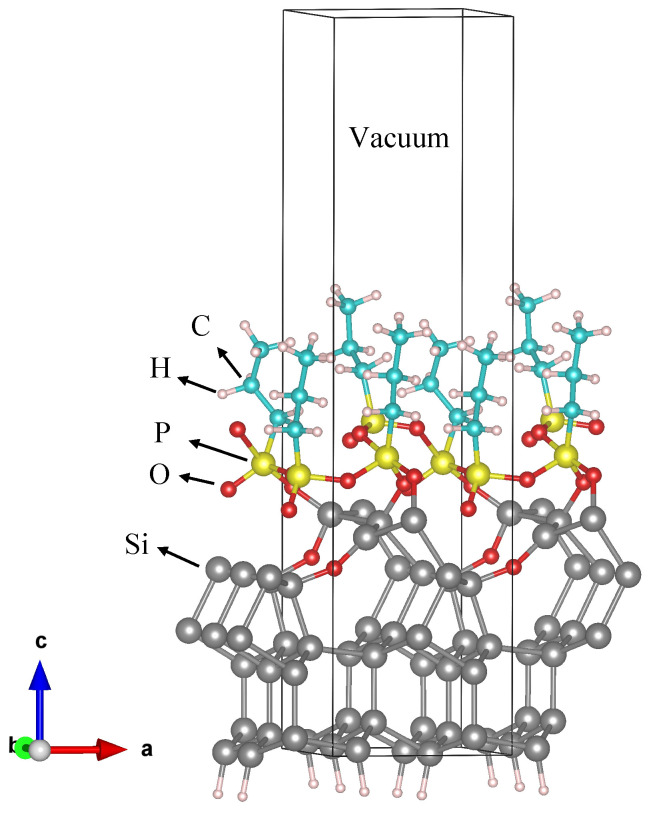
Adsorption model of the grafted DPP molecules (without the aliphatic chains) on a Si (111) 2 × 1 reconstructed surface after DFT geometry optimization.

**Figure 6:**
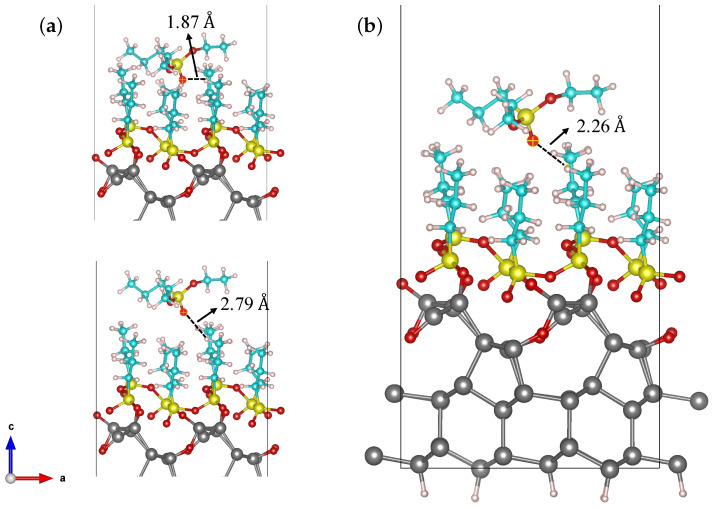
(**a**) The initial DPP positions including shorter (**top panel**) and longer (**bottom panel**) vertical distances from the first layer and (**b**) the most stable structure of physisorbed DPP on a Si (111) 2 × 1 reconstructed surface covered by a broken DPP layer.

Figure 5 shows the optimized geometry of the molecules grafted on a Si (111) surface. As shown in the figure, the molecule assembles itself in packeted groups, which are chemically bonded with each other through P-O-P bonds and with the Si substrate through P-O-Si bonds, with an oxygen atom embedded into the Si surface. In principle, after the optimization process, the molecules located at the valley of the silicon surface were lifted to bond to the next molecules through oxygen. Our calculations reveal that the Si (111) surface structure was deformed due to the strain induced by the significant difference in atomic radius between embedded O (48 pm) and Si (111 pm) atoms. The longest Si-O bond forms with a length of only 1.78 Å, suggesting strong chemical bonds forming. Because of the strong adsorption between broken DPPs and Si atoms, the Si atoms were forced to move away from their initial positions. By comparing the DFT simulation results with the thinnest part of the branches in Figure 4, we can estimate the number of molecules arranged in the thickness of the branch: since the simulated Si cell is 6.60 Å wide and there are two molecules per Si cell side, the thinnest part of the branch (8 nm thick) is made of about 24 molecules in a row. To evaluate the formation of the second molecule layer, we put the entire DPP molecule (28 atoms) on top of the first molecular layer. The optimization process was carried out for positions with different vertical distances between DPP and the first layer of molecules (Figure 6). As shown in Figure 6, the shortest distance between the second molecular layer and the first one is 2.26 Å which is a relatively large distance indicating a weak interaction between the second layer of DPP and the first one. All the bond lengths of the second DPP layer show negligible changes before and after adsorption, indicating that a small charge transfer occurs between DPP and the first molecular layer. These results demonstrate the weak physical adsorption of the DPP molecule over the first molecular layer.

After studying the molecules deposition process, we wanted to investigate the electrical properties of the samples. It is already known that by increasing the deposition time, in the 20% as-deposited samples, i.e., without cleaning, the carrier dose and the junction depth increase monotonically, until a saturation condition is reached (at 100–150 min) [10]. In this paper, we present the electrical measurements of carrier concentration profiles at 150 min, i.e., in the saturation conditions where the full layer is deposited and the complete coverage is obtained, both for the as-deposited and cleaned conditions and compare the two cases.

**Figure 7:**
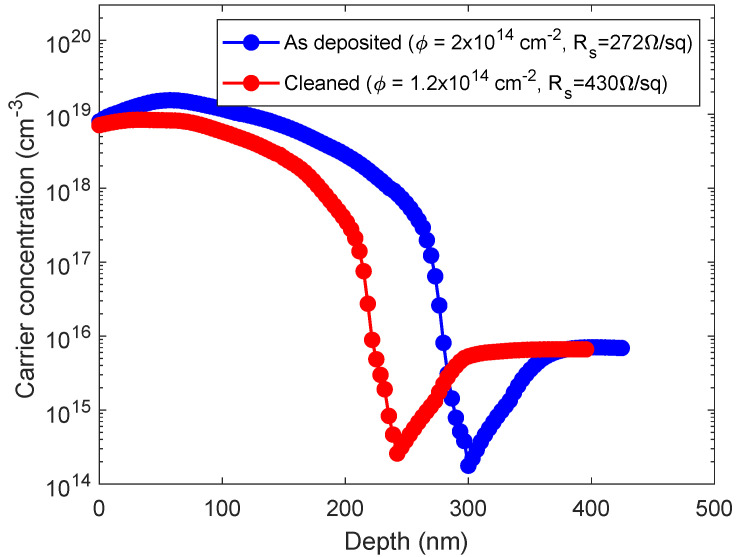
Carrier concentration profile of the as-deposited (blue line) and cleaned (red line) samples with 150 min deposition in a 10% concentrated solution.

After the molecules deposition, we treated the samples with a thermal annealing at 1050 °C for 500 s to diffuse the dopant inside the Si substrate. Figure 7 shows the electrically active carrier concentration profiles, obtained by SRP, of <111> Si substrates dip-coated into a 10% concentrated solution for 150 min, i.e., in the saturation conditions, where the full layer is deposited, and the complete coverage is obtained, in two cases: as-deposited (blue line) and cleaned (red line). The graph shows that both the peak carrier concentration values reach 10${}^{19}$ cm${}^{-3}$ with a junction depth of about 300 nm for the as-deposited case and of about 230 nm for the cleaned one. The carrier dose is 2 × 10${}^{14}$ #/cm${}^{2}$ (with a sheet resistance of 272 Ω/sq) for the former, and 1.2 × 10${}^{14}$ #/cm${}^{2}$ (with a sheet resistance of 430 Ω/sq) for the latter. The reason behind these results could reside in the role of physisorbed molecules in the diffusion process. Since the only difference between the preparation of the two samples is whether or not the samples surfaces have been cleaned, we deduce that the chemisorbed layer and the physisorbed material participate in the diffusion phenomenon. Since a richer dopant source is present on the surface of the as-deposited sample, the dopant dose is higher, and the resulting doping profile is deeper, as per the finite-source impurity diffusion behaviour [44]. By observing the electrical properties of these samples, we can confirm the presence of the molecular over-layers on the sample shown in Figure 1f and of a single layer of chemisorbed molecules on the sample shown in Figure 2f. From our data we can say that the studied doping process, also known in the literature as monolayer doping, can in fact gain further electrical contributions from multiple molecular over-layers.

## 4. Conclusions

We investigated the behaviour of the DPP molecule at several stages of the deposition time during the MD process. The high resolution morphological data on the DPP surface coverage show that: (i) the molecules form a full layer on top of the Si substrate at about 100–120 min of deposition; (ii) a further layer of molecules starts to form over the first one. After cleaning the samples, the coverage decreases and this becomes more evident at long deposition times. By comparing the two sets of coverage data, the as-deposited and the treated ones, we understand that there are covalent, thus strong, bonds between the first layer of molecules and the substrate, while the second layer is attached by weak bonds. To support these understandings, DFT calculations of the first layer of molecules on Si, and of the second layer over the first one have been performed. The simulations show that the DPP molecules of the first layer bond with the substrate through P-O-Si bonds, and with each other through P-O-P bonds. The simulations also indicate the weak nature of the forces between the second and first molecular layer. Electrical measurements performed on the cleaned sample with a full monolayer of molecules on top, demonstrate the efficiency of the doping method, even after cleaning. The comparison between as-deposited and cleaned samples electrical characteristics demonstrates how physisorbed molecules participate in the doping process, and how, with the use of a surface cleaning step, the dopant dose and doping profile can be further controlled. These results will allow for a finer tuning of the electrical properties of many and diverse nano-sized devices, also based on three-dimensional architectures or hollow structures, such as nano-sensors, nano-photodiodes, nano-structured solar cells or fin field-effect transistors.
